# Emotional Distress in Patients With Cancer: A Cross-Sectional Study

**DOI:** 10.1097/jnr.0000000000000694

**Published:** 2025-08-20

**Authors:** Yu-Chi LI, Shu-Ching MA, Hsiu-Hung WANG

**Affiliations:** 1 School of Nursing, College of Nursing, Kaohsiung Medical University, Kaohsiung, Taiwan; 2 Department of Senior Welfare and Services, Southern Taiwan University of Science and Technology, Tainan, Taiwan; 3 Department of Nursing, Chi-Mei Medical Centre, Tainan, Taiwan

**Keywords:** cancer patient, emotional distress, low dignity, demoralization, depression

## Abstract

**Background:**

Emotional distress has long been a central concern for clinical health care professionals when caring for patients with cancer. Emotional distress evaluation is one of the accreditation criteria for cancer centers. The ability to quickly and effectively assess, identify, and treat emotional distress in patients with cancer has become an essential skill for health care professionals.

**Purpose:**

The purposes of this study were to explore the related factors of emotional distress and propose emotional distress score cutoff points to identify the presence of psychological problems or psychiatric disorders in patients with cancer.

**Methods:**

This cross-sectional study was conducted between August 2021 and April 2022 on 400 patients with cancer. The 5-item Brief Symptom Rating Scale (BSRS-5) was used to measure emotional distress in patients with cancer. Data collection employed a structured questionnaire including demographics, BSRS-5, Patient Dignity Inventory-Mandarin version (PDI-MV), Demoralization Scale-Mandarin version (DS-MV), and Patient Health Questionnaire-9 (PHQ-9). Data analysis was performed using SPSS software version 26.0. Data were analyzed using an independent *t* test, one-way analysis of variance, Pearson’s correlation, and receiver operating characteristic curve.

**Results:**

Emotional distress was found to be significantly related to cancer stage (*F*=3.37, *p=*.019), disease characteristics (*t*=2.29, *p=*.023), and treatment phase (*F*=3.12, *p=*.015), with being in stage IV, receiving a recurrence diagnosis, and receiving chemotherapy associated with a higher likelihood of emotional distress. Sensitivity and specificity of the BSRS-5 with an aggregate score of 3.5 or above were, respectively, 74.0% and 84.8% for low dignity (PDI-MV ≥35), 79.1% and 69.6% for demoralization (DS-MV ≥30), 76.7% and 83.8% for depression (PHQ-9 ≥ 10), and 92.0% and 84.0% for suicidal ideation. The BSRS-5 exhibits excellent discrimination for both psychological problems and psychiatric disorders, for example, low dignity, demoralization, depression, and suicidal ideation, in patients with cancer.

**Conclusions/Implications for Practice:**

In this study, emotional distress was shown to be significantly related to demographic factors, including cancer stage, disease characteristics, and treatment phase. Thus, health care professionals should be particularly concerned when patients with cancer are in stage IV, diagnosed with recurrence, or undergoing chemotherapy. Health care professionals should regularly employ brief and highly reliable questionnaires to help evaluate the emotional distress of patients with cancer. Regular evaluation can facilitate the early detection of emotional distress, psychological problems, and psychiatric disorders such as low dignity, demoralization, depression, and suicidal ideation in these patients.

## Introduction

Every year, some 19.3 million new cancer cases are diagnosed, and nearly 10 million cancer-related deaths occur globally (Sung et al., 2021). Cancer incidence, prevalence, and mortality rates are expected to increase significantly through the next 40 years, potentially making cancer the leading cause of death worldwide by 2060 (Mattiuzzi & Lippi, 2019). Regardless of the type of treatment, some degree of emotional distress occurs at every stage of cancer. Studies have exhibited that 46.2%–52.0% of patients with cancer experience emotional distress (Carlson et al., 2019; Mehnert et al., 2018). Emotional distress may affect daily activities and willingness to participate in treatment. Moreover, severe emotional distress can worsen symptoms, affect treatment outcomes, and result in psychological problems, psychiatric disorders, suicidal ideation, or suicidal behavior (Bulotiene & Pociute, 2019). Early evaluation and prevention of emotional distress are necessary for these patients. A short, reliable, and valid questionnaire designed to evaluate emotional distress in patients with cancer that provides excellent discrimination for other psychological problems or psychiatric disorders would greatly benefit both cancer patients and health care professionals in clinical settings.

### Background

Emotional distress in patients with cancer is a key research topic in psycho-oncology, and the ability to evaluate this distress is one of the accreditation criteria for cancer centers (Riba et al., 2019). Common presentations of emotional distress in patients with cancer include sadness, crying, insomnia, hostility, and inferiority as well as severe psychological problems or psychiatric disorders such as low dignity, anxiety, demoralization, depression, suicidal ideation, and suicidal behavior (Cook et al., 2018; Li et al., 2023). Rapid and effective evaluation, identification, and treatment of emotional distress in patients with cancer have become essential skills for health care professionals.

Research on dignity, demoralization, and depression in patients with cancer has increased during the last decade (Hong et al., 2022; Lindwall & Lohne, 2021; Riedl & Schuessler, 2022). Dignity is a basic human need, with everyone hoping to preserve dignity in their environments and situations, including during disease treatment at medical institutions (Lindwall & Lohne, 2021). However, frailty and impaired physical function and encountering the threat of death adversely affect patients with cancer, and if health care providers do not meet their needs, patients may experience low-dignity–related emotional distress (Chochinov et al., 2008; Lindwall & Lohne, 2021). Severe low dignity in patients with cancer may manifest in anxiety, demoralization, depression, and even suicidal ideation or behavior (Bovero et al., 2019; Pergolizzi et al., 2021).

Demoralization is a common psychiatric disorder in patients with cancer that has attracted attention in recent years. These patients are at significant risk of demoralization as a result of stress of treatment, physical frailty, changes in personal roles, increased family burden, and emotional distress (Hong et al., 2022; Peng et al., 2021), with demoralization affecting 16.0%–57.6% of cancer patients and a mean prevalence of 35.8% (Gan et al., 2022). Furthermore, demoralization has been correlated with suicidal ideation and suicidal behavior (Liu et al., 2020).

Depression is the most common comorbidity in patients with cancer (Riedl & Schuessler, 2022), with a reported incidence of 11.0%–57.0% (Götze et al., 2020; Jimenez-Fonseca et al., 2018). These patients who are depressed exhibit more severe physical and mental symptoms and have poorer cancer prognoses (Riedl & Schuessler, 2022). Depression increases the risk of adverse mental health outcomes in patients with cancer, such as poor quality of life and functional status and increased suicide and mortality rates (Adjei Boakye et al., 2020).

The aforementioned findings reveal that emotional distress, such as low dignity, demoralization, and depression, can cause suicidal ideation or suicidal behavior in patients with cancer. Therefore, health care professionals must possess knowledge and skills regarding professional evaluation and periodically assess patients with cancer for emotional changes.

In Taiwan, many hospitals regularly use assessment scales to assess the emotional state of patients with cancer and, based on assessment results, a psychiatrist or psychologist may be consulted for treatment options. The five-item Brief Symptom Rating Scale (BSRS-5) is a tool commonly utilized in the emotional evaluation of patients with cancer in Taiwan (Chen et al., 2022). However, the relationship between the BSRS-5 and psychological problems or psychiatric disorders such as low dignity, demoralization, depression, and suicidal ideation and the related cutoff scores has rarely been addressed in the literature in the context of patients with cancer. Thus, this study was designed to explore the related factors of emotional distress and propose cutoff scores for the BSRS-5 score for identifying low dignity, demoralization, depression, and suicidal ideation in patients with cancer to provide a reference for health care professionals.

## Methods

### Study Design, Settings, and Participants

A cross-sectional design and convenience sampling were employed, and study data were collected from patients undergoing cancer treatment at nine medical and surgery wards at one medical center in southern Taiwan. The participants were recruited between August 2021 and April 2022. Inclusion criteria were: (1) diagnosis of cancer confirmed by the attending physician; (2) at least 20 years old; (3) undergoing cancer treatment as an inpatient; and (4) voluntary participation after understanding the study purposes and procedures and signing informed consent. Exclusion criteria were: (1) diagnosis of dementia, delusion, or other brain organic disease, and (2) having impaired consciousness or being incapacitated. The minimum sample size of 343 was calculated using G-power 3.1.9.4 based on a two-tailed, small effect size of 0.15, alpha level of .05, and power (1−β) of 0.80 (Cohen, 2013; Tsou, 2022). Of the 442 patients approached, 400 met the inclusion criteria and were enrolled as participants. All of the tests were reported on a two-tailed basis and *p* < .050.

### Instruments

#### Demographic characteristics

Demographic data collected included age, gender, marital status, children, educational level, employment, cohabitation status, religious beliefs, tumor site, cancer stage, disease characteristics, and treatment phase.

#### Emotional distress

The BSRS-5 was used to measure the level of emotional distress over the past week. This scale is a self-report questionnaire with five items measuring insomnia, anxiety, hostility, depression, and inferiority, respectively, and an additional sixth question: “Do you have any suicide ideation?” (Lee et al., 2003). The five items of the BSRS-5 are each rated on a five-point Likert scale (from 0=*not at all* to 4=*extremely*), with higher scores indicating greater concern. A total BSRS-5 score ≥6 indicates the presence of emotional distress, and a score >14 or a score of more than 1 on the additional suicidal ideation survey item indicates risk of suicidal behavior. The BSRS-5 earned a Cronbach’s α of .77–.89 in various populations, indicating its validity and reliability (Sanjaya et al., 2023; Tsou, 2022), and the Cronbach’s α in this study was .87.

#### Low dignity

The original English version of the Patient Dignity Inventory (PDI) was designed to measure the level of dignity in patients with cancer during the most recent few days (Chochinov et al., 2008). The PDI Mandarin version (PDI-MV), translated by Li et al. (2018), includes the four dimensions of existential distress, loss of support and sense of meaning, symptom distress, and loss of autonomy. The PDI-MV is a self-report questionnaire with a total of 25 items, each scored using a five-point Likert scale (from 1=*not a problem* to 5=*an overwhelming problem*). A total score of ≥35 indicates low dignity (Li et al., 2023). Cronbach’s α values for the PDI-MV and its four dimensions were .95 and .83–.95 (Li et al., 2018), respectively, and the Cronbach’s α of the PDI-MV in this study was .93.

#### Demoralization

The original English version of the Demoralization Scale (DS) was designed to assess demoralization level during the past 2 weeks (Gan et al., 2022). The DS Mandarin version (DS-MV), translated by Hung et al. (2010), includes the five dimensions of loss of meaning, dysphoria, disheartenment, helplessness, and sense of failure. It is a self-report questionnaire with a total of 24 items, each of which is scored using a five-point Likert scale (from 0=*strongly disagree* to 4=*strongly agree*) and a total score of ≥30 indicating demoralization (Hung et al., 2010). Cronbach’s α values for the DS-MV and its five dimensions were .92 and .69–.88 (Hung et al., 2010), respectively, and the Cronbach’s α value of the DS-MV in this study was .92.

#### Depression

The original English version of the Patient Health Questionnaire-9 (PHQ-9) was designed to assess the level of depression over the past 2 weeks (Kroenke et al., 2001). The Mandarin version of the PHQ-9, translated by Liu et al. (2011), is a self-report questionnaire with a total of nine items, each of which is scored using a four-point Likert scale (from 0=*not at all* to 3=*almost every day*). A total score of ≥10 has a sensitivity of 86% and specificity of 94% for depression. The Cronbach’s α of the PHQ-9 was .80 in Liu et al. (2011) and was .86 in this study.

### Data Collection

The study protocol was approved by the institutional review board of Chi Mei Medical Centre (approval no. 11005-014). Informed consent and confidentiality were obtained from all of the participants. Permission to use the BSRS-5, PDI-MV, DS-MV, and PHQ-9 was obtained. Permission to contact the participants and conduct the study was obtained from a medical center in Southern Taiwan. The participants were recruited through face-to-face interviews with a researcher, and data were collected using a self-report questionnaire with standard instructions. The researcher is a licensed nurse with more than 20 years of clinical nursing experience who has previously conducted many questionnaire survey studies. The researcher checked the list of cancer patients hospitalized in the ward every day, screened patients who met the inclusion criteria, confirmed their cognitive abilities with the attending physician, and then recruited eligible patients face-to-face. The researcher elucidated the purpose and procedures of the study to these patients, obtained their written informed consent, and then conducted the questionnaire survey. The participants had the option of completing the questionnaire on their own or through a one-on-one interview with the researcher. If a participant had questions, the researcher explained on the spot and assisted them with completing the questionnaire.

A total of 442 questionnaires were conducted for this study, and 400 were completed (response rate: 90.5%). The participants took ~20 min to complete the survey. To adhere to research ethics and respect the participants, the researcher did not ask the reasons why 42 of the participants did not complete the survey questionnaire.

Data from the questionnaire were number coded and stored by the study team. Non-study team members were not permitted to view the questionnaire data. Participants had the right to withdraw from the study at any time. The research assistant provided appropriate support to participants if needed, informed the attending physician to assist with management, and provided counseling as well as professional assistance.

### Statistical Analysis

After data coding, statistical analyses were performed using SPSS version 26.0 (IBM Corp., Armonk, NY, USA). Descriptive statistical analyses were performed using frequencies, scores, percentages, means, and standard deviations. Inferential statistical analyses were performed using the independent *t* test, one-way analysis of variance, Pearson’s correlation, and receiver operating characteristic (ROC) curve.

## Results

### Participant Demographics and Clinical Characteristics

The 400 participants comprised 219 males (54.8%) and 181 females (45.3%) of a mean age of 60.00 (*SD*=12.34) years. Most were initial diagnosis patients (63.7%) and were being treated using two cancer treatment types (33.3%). The demographics and clinical characteristics of the participants are shown in Table 1.

**Table 1:** Demographics Participants and Correlations and Mean Difference Between Demographics With the Five-Item Brief Symptom Rating Scale (N=400)

Variable	*n* (%)	*M*±*SD*	*r/F*/*t*	*p*	Post Hoc
Age (year; range; *M*±*SD*)	20–95	60.00±12.34	*r=*.01	.872	
20–34	8 (2.0)	3.63±2.77	*F*=0.48	.750	
35–49	73 (18.3)	3.78±3.15			
50–64	164 (41.0)	4.13±3.63			
65–79	133 (33.3)	4.02±3.63			
≥80	22 (5.5)	4.91±3.93			
Gender			0.76	.450	
Male	219 (54.8)	3.94±3.61			
Female	181 (45.3)	4.21±3.47			
Marital status			0.12	.908	
Married	255 (63.7)	4.05±3.51			
Single	145 (36.3)	4.09±3.61			
Children			0.35	.729	
No	48 (12.0)	3.90±3.65			
Yes	352 (88.0)	4.09±3.53			
Education			1.13	.261	
College or above	81 (20.2)	3.67±3.44			
Below college	319 (79.8)	4.16±3.57			
Employment			1.74	.082	
Yes	200 (50.0)	3.76±3.39			
No	200 (50.0)	4.37±3.66			
Cohabitation status			0.74	.460	
Live with family	379 (94.8)	4.03±3.52			
Alone	21 (5.2)	4.62±4.04			
Religious beliefs			0.69	.494	
No	3 (0.7)	2.67±2.31			
Yes	397 (99.3)	4.07±3.55			
Tumor site			*F*=1.08	.375	
Lung	26 (6.5)	4.15±3.31			
Liver	55 (13.8)	3.47±3.68			
Digestive tract	107 (26.8)	3.70±3.10			
Breast	60 (15.0)	4.30±3.38			
Head and neck	33 (8.3)	5.27±3.97			
Prostate	7 (1.8)	2.43±2.15			
Pancreas	12 (3.0)	5.42±4.33			
Gastric	4 (1.0)	4.50±3.11			
Esophagus	13 (3.3)	3.46±2.79			
Ovarian	5 (1.3)	5.20±2.68			
Cervix	2 (0.5)	2.00±1.41			
Other (multiple tumor sites)	76 (19.0)	4.25±4.11			
Cancer stage			*F*=3.37	.019	
① I	93 (23.3)	3.09±3.31			① < ④
② II	81 (20.2)	4.11±3.37			
③ III	111 (27.8)	4.34±3.68			
④ IV	115 (28.7)	4.55±3.59			④ > ①
Disease characteristics			2.29	.023	
Initial diagnosis	300 (63.7)	3.83±3.33			
Recurrence diagnosis	100 (36.3)	4.76±4.06			
Treatment phase			*F*=3.12	.015	
① Surgery	91 (22.8)	3.07±3.33			① < ③
② Radiotherapy	2 (0.5)	4.50±0.71			
③ Chemotherapy	122 (30.5)	4.67±3.88			③ > ①
④ 2 treatments (①+②; ①+③ or ②+③)	133 (33.3)	3.96±3.23			
⑤ 3 treatments (①+②+③)	52 (13.0)	4.62±3.59			
PDI-MV (range; *M*±*SD*)	25–94	36.06±12.72	*r=*.72	<.001	
Scoring <35	250 (62.5)	2.39±2.20	15.40	<.001	
Scoring ≥35	150 (37.5)	6.85±3.60			
DS-MV (range; *M*±*SD*)	4–76	28.36±14.28	*r=*.63	<.001	
Scoring <30	247 (61.8)	2.62±2.46	12.14	<.001	
Scoring ≥30	153 (38.2)	6.40±3.77			
PHQ-9 (range; *M*±*SD*)	0–26	5.84±4.62	*r=*.78	<.001	
Scoring <10	327 (81.8)	3.07±2.58	14.65	<.001	
Scoring ≥10	73 (18.3)	8.49±3.89			
Suicidal ideation (range; *M*±*SD*)	0–4	0.45±0.83	*r=*0.83	<.001	
No	288 (72.0)	2.43±1.94	21.93	<.001	
Yes	112 (28.0)	8.26±3.26			
BSRS-5 (range; *M*±*SD*)	0–18	4.06±3.54			
Scoring <6	291 (72.7)				
Scoring ≥6	109 (27.3)				

*Note*. Means and *SD*s refer to scores on the five-item brief symptom rating scale.

BSRS-5 = 5-item brief symptom rating scale; DS-MV = demoralization scale Mandarin version; PDI-MV = patient dignity inventory Mandarin version; PHQ-9 = patient health questionnaire-9.

### Relationships Among Demographics, PDI-MV, DS-MV, PHQ-9, Suicidal Ideation, and BSRS-5

The mean scores for the BSRS-5, PDI-MV, DS-MV, and PHQ-9 and for suicidal ideation were 4.06±3.54, 36.06±12.72, 28.36±14.28, 5.84±4.62, and 0.45±0.83, respectively, with scores ranging from 0–18, 25–94, 4–76, 0–26, and 0–4, respectively. There were 109 (27.3%), 150 (37.5%), 153 (38.2%), 73 (18.3%), and 112 (28.0%) participants assessed with emotional distress (BSRS-5 ≥6), low dignity (PDI-MV ≥35), demoralization (DS-MV ≥30), depression (PHQ-9 ≥10), and suicidal ideation, respectively.

Cancer stage (*F*=3.37, *p=*.019), disease characteristics (*t*=2.29, *p=*.023), and treatment phase (*F*=3.12, *p=*.015) were found to relate significantly to the BSRS-5. Specifically, patients in stage IV cancer, who had a recurrence diagnosis, and who were receiving chemotherapy were associated with higher mean BSRS-5 scores and thus more likely to be affected by emotional distress.

Furthermore, PDI-MV (*r=*.72, *p*<.001), DS-MV (*r=*.63, *p*<.001), PHQ-9 (*r=*.78, *p*<.001), and suicidal ideation (*r=*.83, *p*<.001) were found to correlate positively and relate significantly with BSRS-5 score (Table 1). This indicates that participants with low dignity (*t*=15.40, *p*<.001), demoralization (*t*=12.14, *p*<.001), depression (*t*=14.65, *p*<.001), or suicidal ideation (*t*=21.93, *p*<.001) faced a higher likelihood of emotional distress. Other variables were not found to relate significantly to emotional distress (Table 1).

### Cutoff Point of the BSRS-5

**Figure 1:**
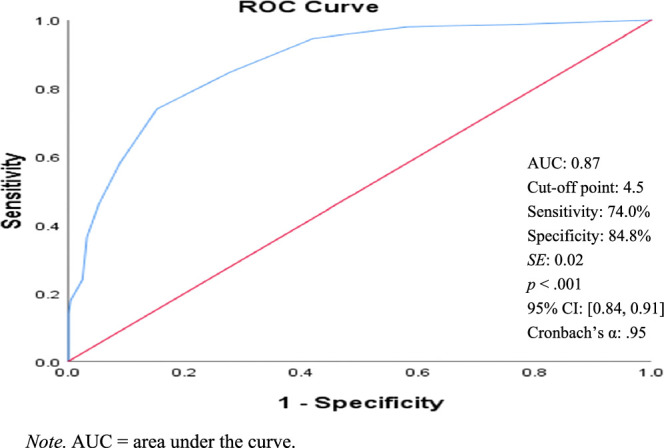
Receiver Operating Characteristic (ROC) Curves of the Five-Item Brief Symptom Rating Scale for Low Dignity (Patient Dignity Inventory-Mandarin Version ≥35)

**Figure 2:**
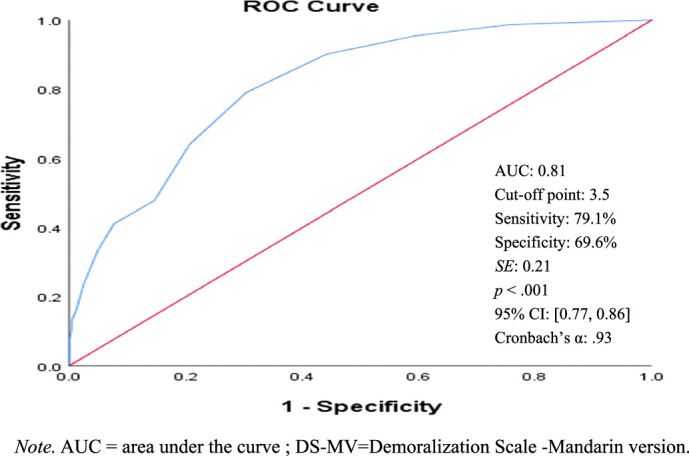
ROC Curves of BSRS-5 for Demoralization (DS-MV ≥30)

**Figure 3:**
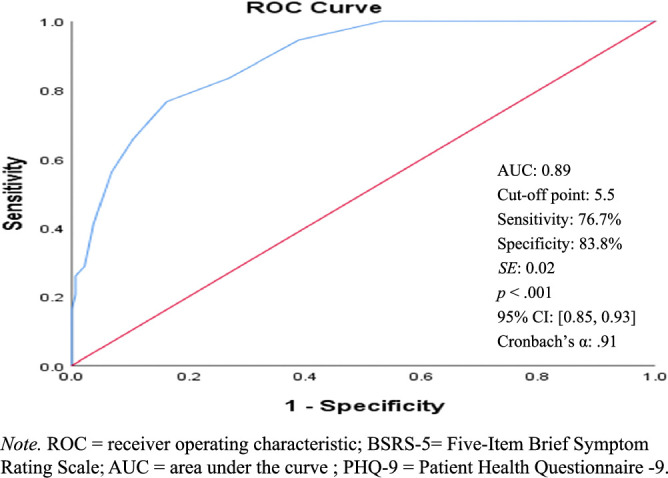
ROC Curves of BSRS-5 for Depression (PHQ-9 ≥10)

**Figure 4:**
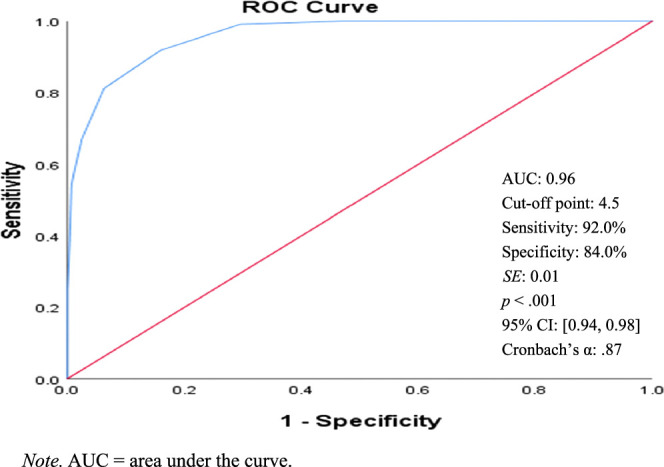
ROC Curves of BSRS-5 for Suicidal Ideation

As shown in Figures 1–4, the ROC curve and area under the curve (AUC) of the BSRS-5 (total score) detect low dignity, demoralization, depression, and suicidal ideation. The findings indicate that using a BSRS-5 cutoff point of 3.5 obtained an AUC, sensitivity, and specificity for demoralization (DS-MV ≥30) of 0.81, 79.1%, and 69.6%, respectively. When this was raised to 4.5, the AUC, sensitivity, and specificity for low dignity (PDI-MV ≥35) were 0.87, 74.0%, and 84.8%, and those for suicidal ideation were 0.96, 92.0%, and 84.0%, respectively. When the cutoff was further raised to 5.5, the AUC, sensitivity, and specificity for depression (PHQ-9 ≥10) were 0.89, 76.7%, and 83.8%, respectively. The result indicates that the BSRS-5 exhibits excellent discrimination for low dignity, demoralization, depression, and suicidal ideation in patients with cancer.

## Discussion

This study was designed to explore the related factors of emotional distress and to propose suitable cutoff points for emotional distress scores to accurately identify psychological problems and psychiatric disorders in patients with cancer.

In terms of factors related to emotional distress, the results showed that participants in cancer stage IV and those with a recurrent diagnosis had significantly higher levels of emotional distress compared, respectively, with their peers in stage I and with an initial diagnosis. Fear of disease progression is the most distressing issue for cancer patients (Herschbach et al., 2020). The findings of a previous study and a systematic review indicate that patients with advanced cancer had the highest levels of emotional distress (Cook et al., 2018; Mehnert et al., 2018), which is similar to the findings of this study. Although the relationship between cancer stage and emotional distress is nonlinear, cancer progression may exacerbate emotional distress in patients with cancer (Mehnert et al., 2018). Thus, health care professionals must show more emotional concern and support. In addition, the results of this study showed participants undergoing chemotherapy had significantly greater emotional distress than those who had received surgery. Thus, while chemotherapy may improve cancer survival rates, it has many side effects, including physical effects such as fatigue, insomnia, nausea, and vomiting, and psychological effects such as fear, anxiety, depression, and sadness due to uncertainty about treatment outcomes and disease recurrence (Wagland et al., 2016). Health care professionals should prepare the knowledge and abilities necessary to minimize the side effects of chemotherapy to ameliorate emotional distress in their patients with cancer.

In this study, demographic variables were not found to relate significantly to emotional distress. Notably, previous studies have reported a significant effect of gender on emotional distress in patients with cancer, with females experiencing significantly greater emotional distress than males (Herschbach et al., 2020; Mehnert et al., 2018). Also, some studies have shown older age (≥60 years) to relate significantly and positively with emotional distress (Mehnert et al., 2018), while others have found the opposite, with younger patients expressing significantly greater emotional distress than their older peers with cancer (Carlson et al., 2019; Herschbach et al., 2020). Although gender and age were not found to relate significantly with emotional distress in this study, the average scores on the BSRS-5 show that females had higher levels of emotional distress than males and that emotional distress levels increase with age. The results for factors related to emotional distress differed from previous studies. This is potentially attributable to the different measurement tools used, the mean level of disease severity, and cultural differences. In most prior studies, the Distress Thermometer (Carlson et al., 2019; Herschbach et al., 2020; Mehnert et al., 2018) was used to assess emotional distress in patients with cancer. Moreover, previous studies used large databases with sample sizes of 3,000 to 21,000 in Western countries that included cancer patients in emergency, inpatient, outpatient, and home care settings both during and after treatment. All of the 400 participants in this study were inpatients and currently receiving cancer treatment. However, both this and prior studies agree that patients with cancer all suffer from emotional distress. More research is needed to confirm the gender and age effects on emotional distress in this patient population.

A BSRS-5 score of 6 or above indicates the presence of emotional distress (Sanjaya et al., 2023; Tsou, 2022). In this study, 109 (27.3%) of the participants met this threshold. Furthermore, psychological problems or psychiatric disorders, such as low dignity, demoralization, depression, and suicidal ideation, were found in this study to relate significantly to emotional distress (Bulotiene & Pociute, 2019; Cook et al., 2018). Previous studies have revealed that patients with cancer with a dignity score ≥35 may be affected by low dignity (Li et al., 2023). In this study, the mean dignity score of 36.06 (*SD*=12.72) met the threshold for low dignity. Of the 400 participants, 150 (37.5%) had a PDI-MV score ≥35 points. This finding is similar to those of studies reporting 18.8%– 49.4% of patients with cancer experience dignity-related distress (Bovero et al., 2018; Wang et al., 2023). Accordingly, more attention should be paid to the dignity of patients with cancer in the health care domain and environment. Patient dignity may be directly threatened when health care professionals regard them as terminal patients. Health care professionals exhibiting this type of pessimistic attitude will be unable to provide dignified care to their patients. Health care professionals should possess accurate perspectives and competencies to maintain patient dignity; utilize dignified language, expression, attitudes, and behaviors (Lindwall & Lohne, 2021); respect the autonomy of patients with cancer; increase their self-worth; control physical and psychosocial factors as well as painful experiences that threaten their dignity; and formulate appropriate measures (Bagherian et al., 2020; Bylund-Grenklo et al., 2019).

Of the 400 participants, 153 (38.2%) had demoralization (DS-MV ≥30), 73 (18.3%) had depression (PHQ-9 ≥10), and 112 (28.0%) had suicidal ideation. Previous studies have highlighted that 13.5%–49.4% of patients with cancer experience demoralization (Wang et al., 2023), 14.5%–37.1% have depression (Naser et al., 2021), and 0.7%–46.3% have suicidal ideation (Kolva et al., 2020), echoing the findings of this study. Gan et al. (2022) reported the demoralization score of patients with cancer to be higher than that of those without cancer. Riedl and Schuessler (2022) found the prevalence of depression in patients with cancer to be three to four times higher than in patients without cancer. Demoralization and depression are independent risk factors for suicidal ideation and suicidal behaviors in patients with cancer (Costanza et al., 2022). Therefore, hospitals should actively include demoralization evaluation and depression screening in regular cancer treatments and strive to minimize these factors in patients with cancer to prevent suicidal ideation and behavior.

The findings of this and earlier studies support emotional distress (e.g., demoralization, depression, and suicidal ideation) in patients with cancer as an important topic of global interest. Health care professionals should employ evaluation tools effectively and periodically evaluate emotional changes in patients with cancer to prevent the adverse effects of emotional distress. Although the PDI-MV, DS-MV, and PHQ-9 are suitable for patients with cancer, they comprise over 20 questions, which are time-consuming to complete based on the respondent’s level of understanding.

In Taiwan, the BSRS-5 is widely utilized to evaluate the emotional state of inpatients. In studies, Cronbach’s α for the BSRS-5 was .80 in the general population (Lee et al., 2010), .83 among inpatients (Lee et al., 2021), and .77–.89 among clinical staff (Sanjaya et al., 2023; Tsou, 2022). The Cronbach’s α for the BSRS-5 was .87 in this study. These findings indicate the BSRS-5 has good reliability in various populations. In this study, BSRS-5 scores of 3.5–5.5 were used to obtain sensitivity (79.1%–92.0%) and specificity (69.6%–84.8%) for low dignity, demoralization, depression, and suicidal ideation in patients with cancer, with an AUC range of 0.81–0.96, indicating excellent discrimination. In prior studies, BSRS-5 scores of 3 and 4 have been used as a cutoff point for predicting suicidal ideation, with a sensitivity of 83.0% and specificity of 86.0% (Lee et al., 2010). However, studies on the cutoff point prediction for other types of emotional distress (such as low dignity, demoralization, and depression) are rare. This represents a major difference between this and prior studies and is a contribution of this study to the literature. Patients with cancer encounter the threat of death, and their emotional distress is more complex than that of the general population or non-cancer patients. Periodic evaluation of cancer patients by health care professionals utilizing the BSRS-5 may be expected to expand the availability of care routes suitable for cancer patients. For example, a score ≤3 points should raise nurses’ attention, while a score of 4 should trigger a visit from a spiritual caregiver or clinical psychologist and a score ≥5 should trigger a referral with a psychiatrist or psychotherapist. A scale suitable for the periodic emotional evaluation of patients with cancer should also be developed.

The BSRS-5 proved easy for clinical nurses to implement due to its five-item structure and the need for only an elementary school level of literacy for self-evaluation. Also, the BSRS-5 does not contain sentences on physical symptoms and can avoid the effects of such symptoms on the evaluation of the results. In addition, the BSRS-5 is designed to assess suicidal ideation, showing sensitivity and specificity for low dignity, demoralization, depression, and suicidal ideation in patients with cancer. Therefore, the BSRS-5 is both feasible as well as reliable, and is suitable for detecting premature signs of low dignity, demoralization, depression, and suicidal ideation in patients with cancer.

### Limitations

This study only recruited participants who were inpatients currently receiving cancer treatment. In addition, this study was conducted during the post-COVID-19 epidemic. Thus, whether the emotional distress detected was also affected by current post-COVID-19 conditions should be further explored. Therefore, the results of this study cannot be broadly extrapolated.

### Conclusions

Emotional distress is significantly associated with demographics such as cancer stage, disease characteristics, and treatment phase. Health care professionals should pay more attention to patients with cancer in stage IV, with a recurrence diagnosis, and who are undergoing chemotherapy. The ability to swiftly and accurately assess, identify, and address the emotional distress of patients with cancer is indispensable for health care professionals. Health care professionals should regularly employ a brief and highly reliable questionnaire such as the BSRS-5 to evaluate emotional distress to prevent psychological problems and psychiatric disorders such as low dignity, demoralization, depression, and suicidal ideation in patients with cancer.
